# Employment impacts of the San Francisco sugar-sweetened beverage tax 2 years after implementation

**DOI:** 10.1371/journal.pone.0252094

**Published:** 2021-06-02

**Authors:** Samantha Marinello, Julien Leider, Lisa M. Powell

**Affiliations:** 1 Division of Health Policy and Administration, School of Public Health, University of Illinois Chicago, Chicago, Illinois, United States of America; 2 Institute for Health Research and Policy, University of Illinois Chicago, Chicago, Illinois, United States of America; Universidad Adolfo Ibanez, CHILE

## Abstract

**Introduction:**

Sugar-sweetened beverage (SSB) taxes have been implemented worldwide to raise revenue and reduce consumption of SSBs, which is associated with health harms. Empirical evaluations have found that these taxes are successful at reducing demand for SSBs; however, SSB taxes face opposition, in part because of claims that they will lead to substantial job losses. The purpose of this study is to examine the impact of the San Francisco SSB tax, implemented on January 1st, 2018, on employment.

**Methods:**

Monthly employment counts were obtained from the Bureau of Labor Statistics from January 2013 (5-years pre-tax) through December 2019 (2-years post-tax) for the overall economy, private sector, supermarkets and other grocery stores, convenience stores, limited-service restaurants, and beverage manufacturing. A synthetic control analysis was conducted for each employment outcome. The synthetic controls (i.e., estimated counterfactuals) were generated from a pool of urban control counties using pre-tax labor market-related characteristics.

**Results:**

The synthetic controls had similar labor market-related characteristics and employment outcomes to those in San Francisco in the pre-tax period. Up to 2 years post-tax, differences in employment between San Francisco and the synthetic controls were small and not “statistically significant” based on placebo tests for all employment outcomes.

**Conclusions:**

Up to two years post-tax, we do not find evidence that the San Francisco SSB tax negatively impacted net employment, employment in the private sector, or employment in specific SSB-related industries.

## Introduction

Sugar-sweetened beverage (SSB) taxes are increasingly being implemented as a policy tool to both raise revenue and reduce demand for SSBs given the link between SSB consumption and health harms such as diabetes, obesity, and cardiovascular disease [1–3]. Various forms of SSB taxes (hereafter, SSB taxes refer to taxes on SSBs alone and taxes applied to both SSBs and artificially sweetened beverages) are now present in more than 45 countries worldwide and seven U.S. cities [4, 5]. Numerous evaluations of these taxes have shown that they are effective in reducing the demand for SSBs [6–8]: a recent meta-analysis of SSB tax evaluations conducted in the Region of the Americas assessing price responsiveness of demand for SSBs estimated that a SSB tax that raises prices by 20% is associated with a 27% reduction in demand [5].

Nonetheless, policymakers face substantial opposition to SSB taxes. One such oppositional argument, which has also been made against tobacco and alcoholic beverage taxes, is that these taxes will lead to considerable job loss [7]. Industry and other stakeholders argue that lower sales of SSBs will result in job loss particularly in the industries involved in the production, distribution, and sale of these products. However, some of the lost sales for taxed SSBs will be made up in part from substitution by consumers to untaxed beverages, often produced by the same companies. In addition, money that would have been spent on taxed beverages will be reallocated to other goods and services, thereby creating new jobs in the economy. Further, government spending from the additional tax revenue will also generate new jobs. Thus, there may indeed be some structural shifts in employment, with losses in some industries and gains in others, but SSB taxes, similar to other health taxes, are not expected to result in overall job losses [7].

According to a recent systematic review of diet-related fiscal policies, the evidence base on the labor market impacts of SSB taxes is limited [9]. Further, the current literature includes a number of industry-funded modeling studies that estimate gross job losses from partial analyses [9]. Non-industry peer-reviewed modeling and empirical studies, on the other hand, have consistently found no net reductions in employment or increases in unemployment [9]. One such study assessed the impact of a hypothetical 20% SSB tax on employment for two U.S. states (California and Illinois) accounting for substitution, income effects, and government spending and found reductions in employment in the beverage industry but no net reductions in private sector or overall employment [10]. With respect to empirical tax evaluation studies, following the implementation of the 2014 Mexico taxes on SSBs and nonessential energy-dense foods, a study found no pre- to post-tax changes in employment in related manufacturing industries and a slight increase in food and beverage store employment [11]. Additionally, the study showed no adverse impact on unemployment [11]. In the U.S., two tax evaluations have assessed labor market outcomes following the introduction of the 2017 Philadelphia, PA, sweetened beverage tax imposed on both SSBs and artificially sweetened beverages. The first study found no statistically significant pre- to post-tax changes in monthly unemployment claims in Philadelphia relative to claims in adjacent counties in industries that might have been negatively impacted by the tax [12]. The second study conducted a synthetic control analysis to evaluate the effect of the tax on the number people employed in the overall economy, the private sector, and industries that sell sweetened beverages and found no negative impact up to 2.5 years post-tax [13].

This study adds to the literature by providing evidence of potential impacts on employment for another U.S. jurisdiction and is the first study, to our knowledge, to do so for San Francisco. The San Francisco SSB tax is a 1-cent per ounce tax levied on distributors of beverages (>25 calories per 12 fluid ounces) with added caloric sweeteners and was implemented on January 1st, 2018. This is also the first study to include an assessment of employment in the beverage and supermarket/grocery store industries following the introduction of a SSB tax in the U.S. Specifically, we apply the synthetic control method (SCM) to estimate the impact of the San Francisco SSB tax on the number of people employed in industries that produce and sell SSBs (including the beverage manufacturing, convenience store, supermarket and other grocery store, and limited-service restaurant industries) as well as on private sector and net total employment up to two years after the tax was implemented.

## Methods

### The synthetic control method

This paper uses the SCM to evaluate the impact of the San Francisco SSB tax on employment two-years post-tax. The method is described in detail in Abadie et al. [14, 15] and is summarized in this section in the context of the research question.

The SCM is used in this study to generate counterfactual employment outcomes for San Francisco, i.e., employment in San Francisco if the tax had not been implemented. The estimated counterfactual, called a synthetic control, is a weighted average of control site outcomes, where weights must be non-negative and sum to one. Control sites with positive weights are selected from a larger set of control sites known as a “donor pool.” To construct a valid counterfactual, the donor pool should consist of sites that (1) have similar labor market-related characteristics to those of San Francisco, (2) did not implement a SSB tax, and (3) were not affected by the San Francisco SSB tax or any other SSB tax. Optimal donor pool weights are selected using a set of predictors that are correlated with the determinants of the outcome (i.e., labor supply and demand), such as gross domestic product, the unemployment rate, and the number of establishments. The most important predictor is the lagged outcome, or employment in the pre-tax period. This predictor captures unobserved determinants of employment and reduces the likelihood of omitted variable bias. Weights are derived by minimizing the differences between the synthetic control and San Francisco in predictor values and the employment outcome (measured in root mean squared prediction error (RMSPE)) over the period prior to tax implementation.

There is strong evidence that the synthetic controls are valid counterfactuals (controlling for all other factors that impact employment unrelated to the SSB tax) when they reproduce similar pre-tax employment trends and predictor values, particularly values of the lagged outcome, to those in the treated site, i.e., San Francisco. We show in the Results section that this is true of the synthetic controls used in these analyses. To assess whether post-tax differences relative to the synthetic control represent a “statistically significant” treatment effect of the San Francisco SSB tax, Abadie et al. [14] propose inferential techniques called placebo tests. The most commonly used test, called an in-space placebo test, involves re-estimating the treatment effect for each site in the donor pool (called a “placebo effect”) and then comparing these effects to the estimated effect in the treated site. Sensitivity analyses are also recommended to evaluate the robustness of the results. Results from placebo tests and sensitivity analyses are reported below in the Results section.

The SCM has two major advantages over difference-in-differences (DID) and controlled interrupted time series (CITS) analyses. The first is that it controls for time-varying unobserved confounders as the synthetic control is created to be similar to the treated site in terms of characteristics that are correlated with the dynamics of the outcome. Imbalance on these characteristics can cause bias in DID or CITS analyses. A second advantage is that the SCM can provide a more objective method of selecting a comparison site. Abadie et al. [14] also recommend against using regression-based inference when assessing the impact of an intervention on an aggregate outcome measured with little uncertainty using a small number of treatment and control units.

### Data and sample

Data on monthly employment counts in the overall economy as well as in key industries that produce and sell SSBs were obtained from the Bureau of Labor Statistics’ (BLS) Quarterly Census of Employment and Wages (QCEW) program from January 2013 (5-years pre-tax) through December 2019 (2-years post-tax). These data are primarily collected from states’ unemployment insurance accounting systems and account for more than 95% of U.S. jobs. County-level data were used, as San Francisco is coterminous with San Francisco County.

Employment was assessed in four industries that may have been negatively impacted by the SSB tax. These industries were identified in the QCEW by their North American Industry Classification System (NAICS) code: beverage manufacturing (NAICS 3121), supermarkets and other grocery stores (NAICS 44511), convenience stores (NAICS 44512), and limited-service restaurants (NAICS 722513) (commonly known as fast-food restaurants); public-sector employment was excluded from these industries. Soft drink manufacturing (NAICS 312111) was also considered for the analysis; however, employment data in this industry were suppressed by the BLS in San Francisco during the study period. QCEW data are suppressed to protect the confidentiality of company-specific information. For example, employment data may not be released if a single company dominates an industry within a geographical area. To estimate the effect of the tax on net employment, total employment (NAICS 10) and private-sector employment (NAICS 10, private ownership) were also included.

The donor pool consisted of urban U.S. counties (and county-level equivalents) that did not implement, or border a county that implemented, a SSB tax during the study period. Bordering counties were excluded because their employment outcomes may have been affected by neighboring taxes. Counties were also removed from the donor pool if they had missing data on any predictors. For a given industry, counties were excluded from the donor pool if they had missing employment data or zero employment for the entire study period. The donor pool was restricted to urban counties because they are more likely to be similar to San Francisco in terms of their labor markets as well as other factors that influence employment over time. For all outcomes except beverage manufacturing, counties were classified as urban if they were designated as large central metros by the National Center for Health Statistics’ 2013 Urban-Rural Classification Scheme. For beverage manufacturing, the donor pool was expanded to include large fringe metros because few large central metros met the study criteria. The final donor pools included 62 counties for total, private sector, and limited-service restaurant employment; 61 counties for supermarket and other grocery store and convenience store employment; and 47 counties for beverage manufacturing employment. A description of how we arrived at our donor pool samples and a list of donor pool counties are provided in S1 Fig and S1 Table, respectively.

A set of county-level predictors were chosen to capture the observed and unobserved determinants of labor supply and demand in the pre-tax period. For each employment outcome, average employment in each pre-tax year was used as the lagged outcome predictor. County-level characteristics included personal income per capita adjusted for cost-of-living, gross domestic product (GDP), unemployment rate, total population, population density, and percentage of prime-age workers (ages 25–54); industry-specific characteristics within counties included number of establishments and industry share, defined as the percentage of all workers that were employed in a given industry. Aside from population density, all non-lagged outcome predictors were averaged over the pre-tax period or were single estimates over the pre-tax period (e.g., American Community Survey 5-Year estimate for 2013–2017). The measure of population density was only available as a single estimate from the 2010 Decennial Census. Detailed information regarding the source of each predictor and how it was computed can be found in S2 Table.

The analysis was conducted in Stata/SE 15.0, using the “synth” [16] and “synth_runner” packages [17].

## Results

Table 1 reports weights assigned to control counties that were used to construct the synthetic controls; all other counties in the donor pools received a weight of zero. Predictor balance between San Francisco and the synthetic control for each employment outcome is shown in Table 2. Across synthetic controls, there is strong balance on lagged outcome predictors, i.e., the average number of people employed in each pre-tax year. In most cases, the synthetic controls had other county and industry-specific characteristics similar to those in San Francisco.

**Table 1:** Counties or county-equivalent entities with non-zero weights for total, private sector, supermarkets and other grocery stores, convenience store, limited-service restaurant, and beverage manufacturing employment synthetic controls.

County/City, State	Total employment	Private sector	Supermarkets and other grocery stores	Convenience stores	Limited-service restaurants	Beverage manufacturing
	Weight	Weight	Weight	Weight	Weight	Weight
Denver, CO	0.451					0.057
Fulton County, GA	0.129		0.065	0.006		
Kings County, NY	0.349	0.260			0.082	
New York County, NY	0.053		0.150	0.109	0.141	
Alexandria City, VA	0.018		0.253		0.464	
Riverside County, CA		0.217				
Mecklenburg County, NC		0.135				
Dallas County, TX		0.021				
Travis County, TX		0.366			0.056	
Hennepin County, MN			0.001	0.206	0.089	0.447
St. Louis City, MO			0.070			
Bronx County, NY			0.002	0.047		0.143
Queens County, NY			0.027			
Oklahoma County, OK			0.194			
Harris County, TX			0.040			
Arlington County, VA			0.198	0.606		
Los Angeles County, CA				0.014		
Tarrant County, TX				0.012		
Suffolk County, MA					0.013	
Essex County, NJ					0.088	
Hamilton County, OH					0.068	
Collin County, TX						0.352

Only counties with non-zero weight are shown. Blank cells correspond to a weight of zero.

**Table 2:** Predictor balance for San Francisco and its synthetic control for total, private sector, supermarket and other grocery store, convenience store, limited-service restaurant, and beverage manufacturing employment.

	Total employment		Private sector		Supermarkets and other grocery stores		Convenience stores		Limited-service restaurants		Beverage manufacturing	
Variables	San Francisco	Synthetic Control	San Francisco	Synthetic Control	San Francisco	Synthetic Control	San Francisco	Synthetic Control	San Francisco	Synthetic Control	San Francisco	Synthetic Control
County-level												
Unemployment rate	3.94	5.34	3.94	5.50	3.94	4.31	3.94	3.71	3.94	4.45	3.94	4.52
Gross domestic product ($ 000,000s)	133,725	104,642	133,725	85,493	133,725	134,534	133,725	116,764	133,725	119,350	133,725	78,023
Adjusted personal income per capita	0.76	0.65	0.76	0.56	0.76	0.77	0.76	0.76	0.76	0.77	0.76	0.65
Population density per square mile	17,179	18,188	17,179	9,935	17,179	15,750	17,179	14,461	17,179	18,103	17,179	6,187
Total population	864,263	1,446,430	864,263	1,820,022	864,263	818,902	864,263	811,443	864,263	831,531	864,263	1,116,082
Prime-age workers (25–54) (%)	52.4	47.2	52.4	44.7	52.4	49.2	52.4	51.2	52.4	49.6	52.4	43.8
Industry-specific												
Employment, 2013	611,717	613,397	516,678	518,046	6,969	6,948	313	314	9,327	9,359	232	224
Employment, 2014	640,378	638,942	544,991	545,635	7,110	7,100	339	346	9,970	9,954	275	286
Employment, 2015	674,646	676,231	575,876	575,545	7,448	7,449	362	363	10,443	10,440	447	431
Employment, 2016	703,188	699,085	605,364	598,618	7,463	7,435	323	326	10,870	10,933	515	536
Employment, 2017	716,917	718,647	617,246	619,753	7,536	7,528	336	338	11,552	11,527	659	648
Industry share (%)	-	-	85.5	84.5	1.09	1.30	0.05	0.06	1.56	1.87	0.06	0.07
Number of establishments	57,924	46,282	56,886	46,659	287	290	69	66	779	814	30	25

**Fig 1:**
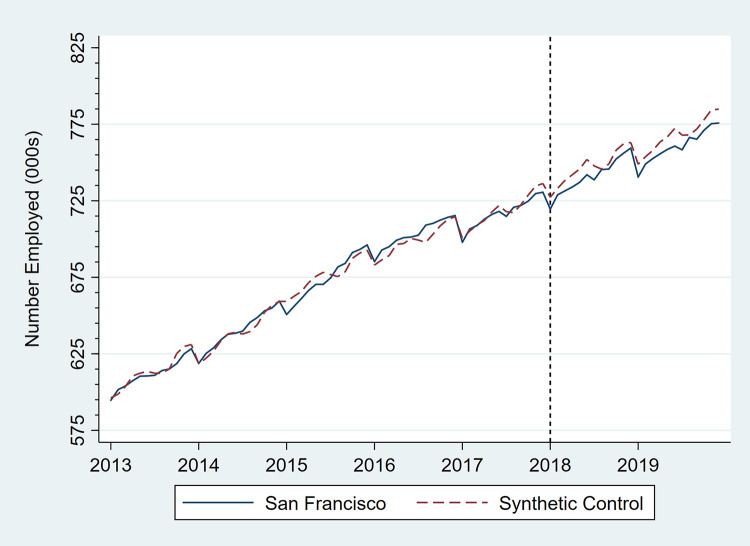
Total number of persons employed, San Francisco, CA, and its synthetic control, monthly, January 2013 through December 2019.

**Fig 2:**
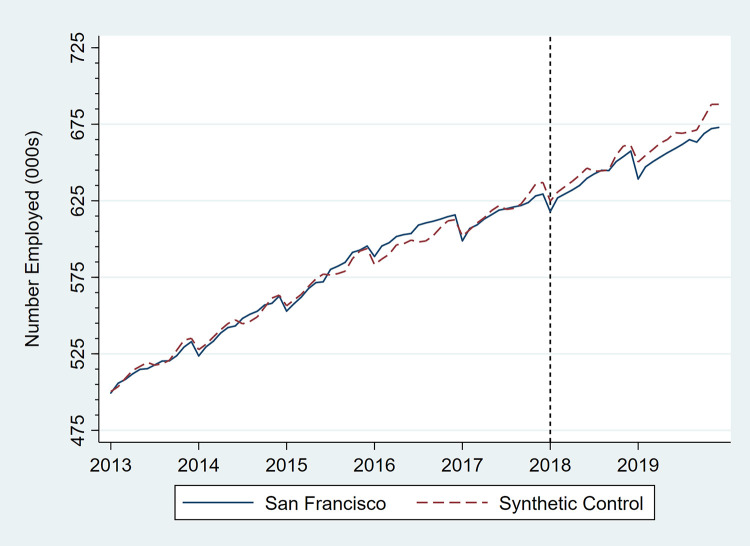
Number of persons employed in the private sector, San Francisco, CA, and its synthetic control, monthly, January 2013 through December 2019.

**Fig 3:**
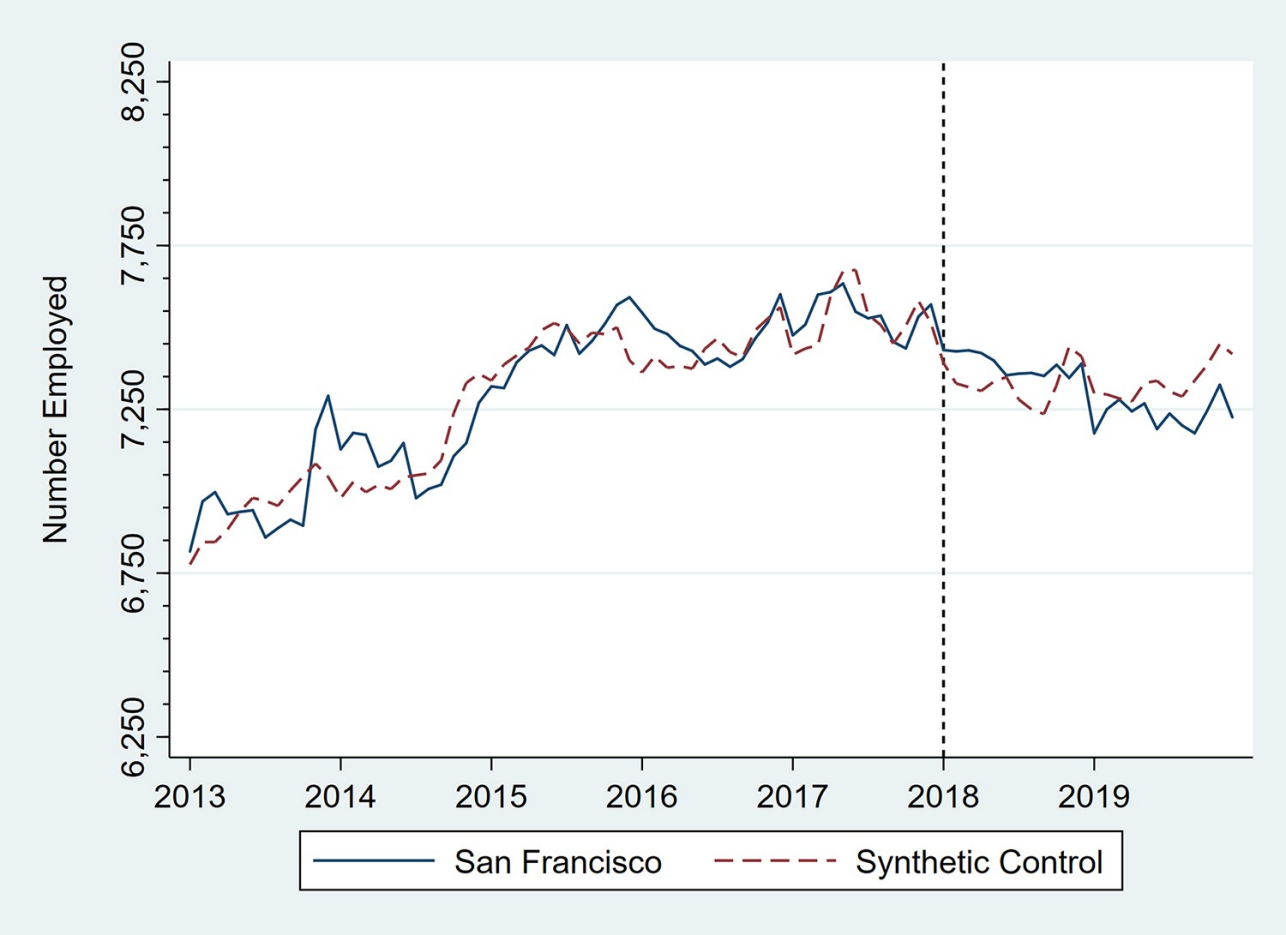
Number of persons employed in supermarkets and other grocery stores, San Francisco, CA, and its synthetic control, monthly, January 2013 through December 2019.

**Fig 4:**
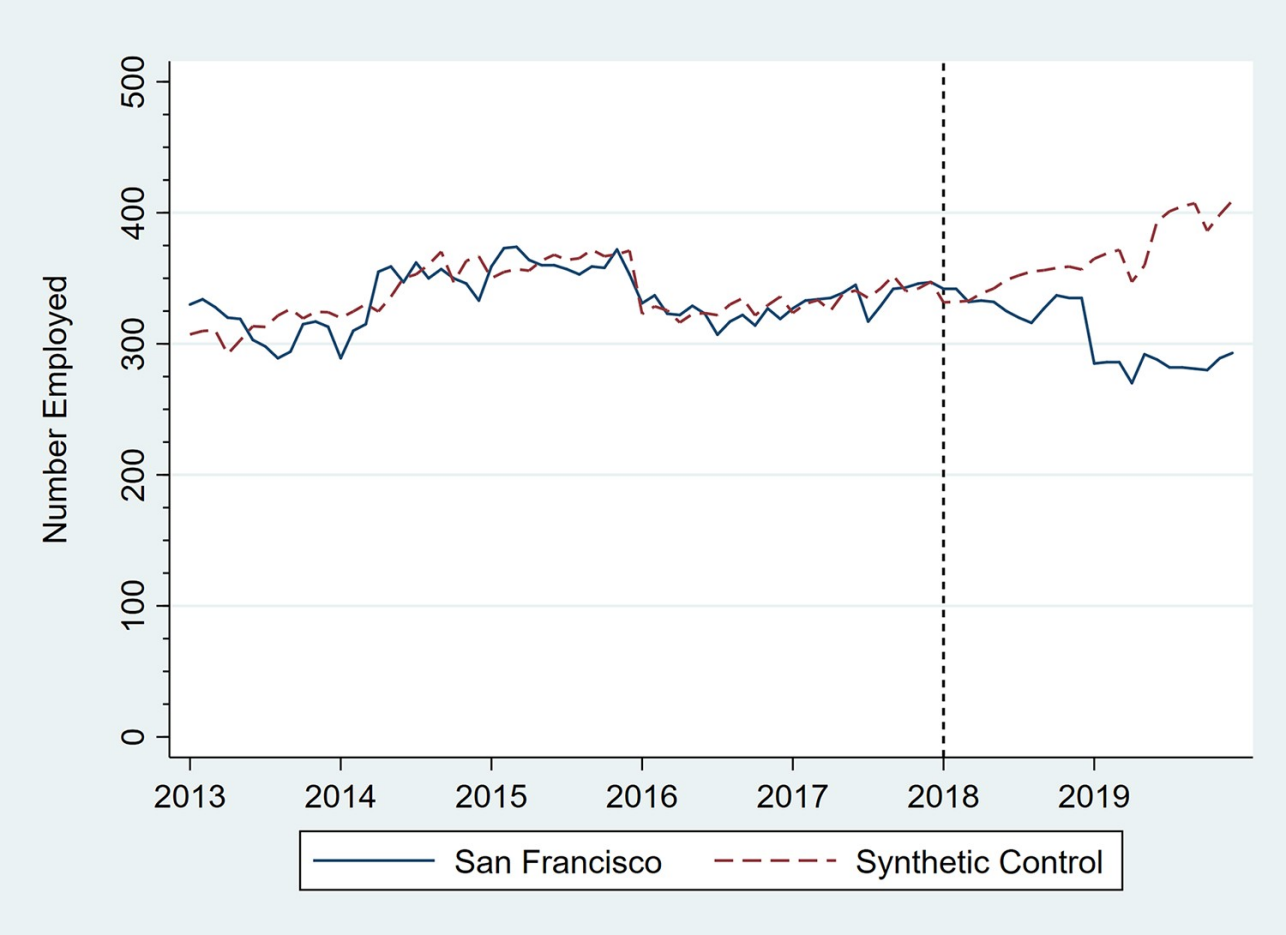
Number of persons employed in convenience stores, San Francisco, CA, and its synthetic control, monthly, January 2013 through December 2019.

**Fig 5:**
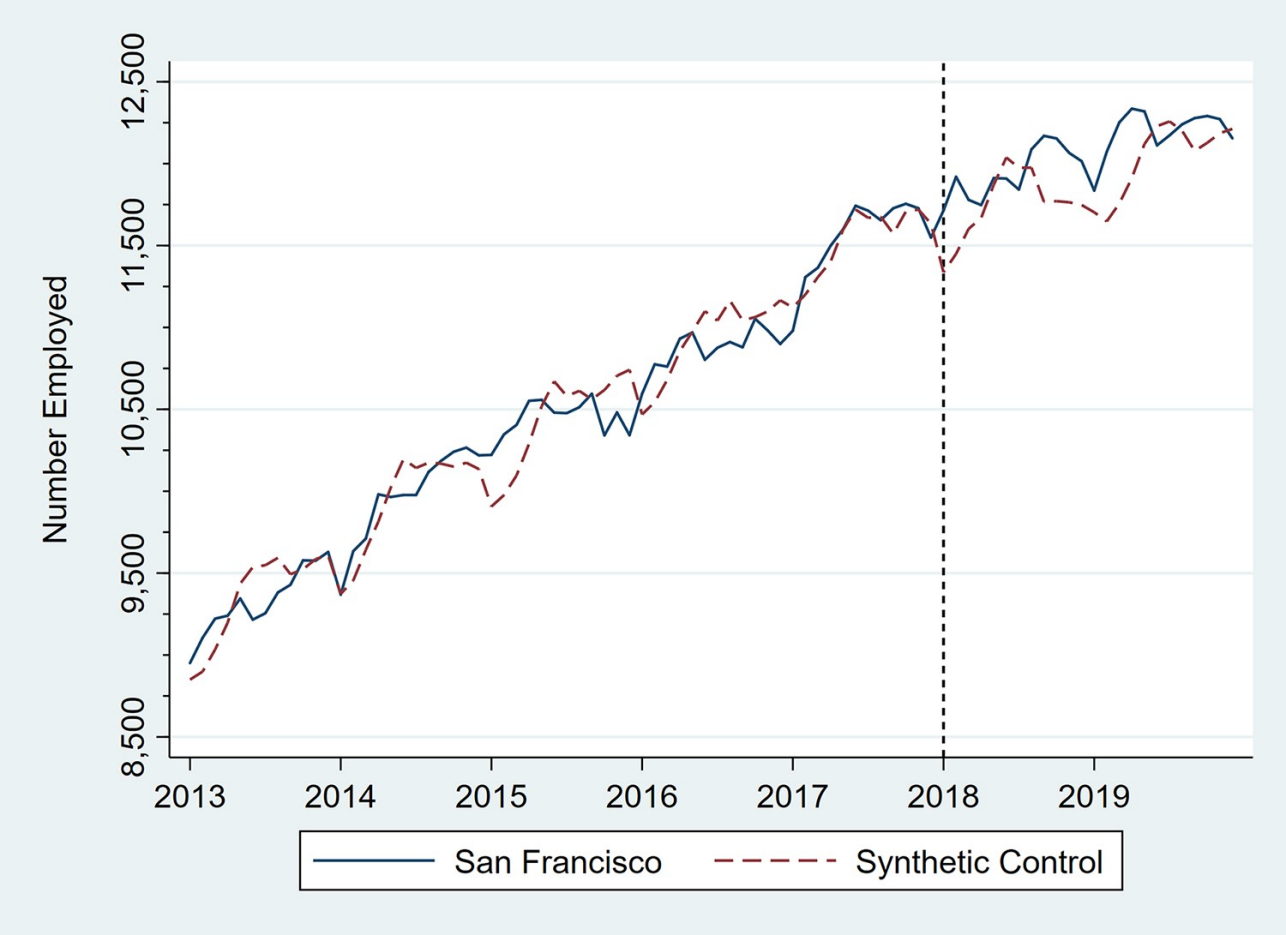
Number of persons employed in limited-service restaurants, San Francisco, CA, and its synthetic control, monthly, January 2013 through December 2019.

**Fig 6:**
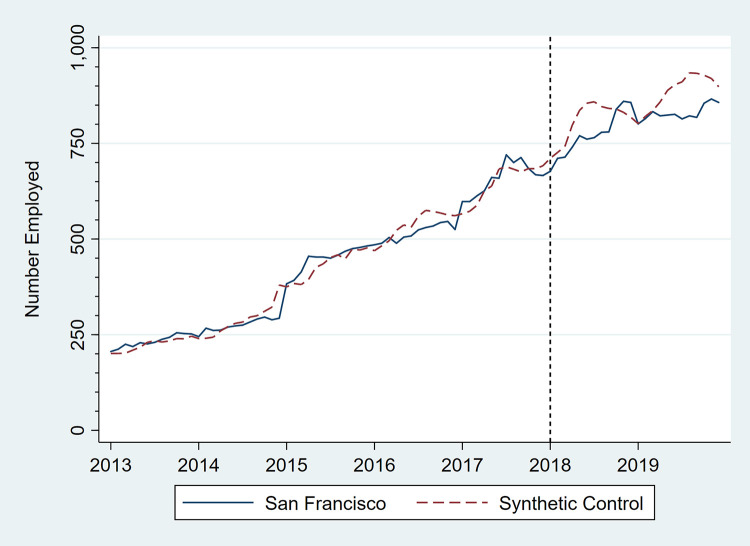
Number of persons employed in beverage manufacturing, San Francisco, CA, and its synthetic control, monthly, January 2013 through December 2019.

Figs 1–6 present monthly employment counts in San Francisco and the synthetic controls for each employment outcome from January 2013 through December 2019. The dashed line at January 2018 indicates the date the tax went into effect. For each employment outcome, the synthetic controls produced very similar employment trajectories to those in San Francisco in the pre-tax period, supporting their validity as counterfactuals. Estimates of the tax impact, given by the differences between San Francisco and synthetic control employment in the post-tax period, show that the differences continue to be small.

**Fig 7:**
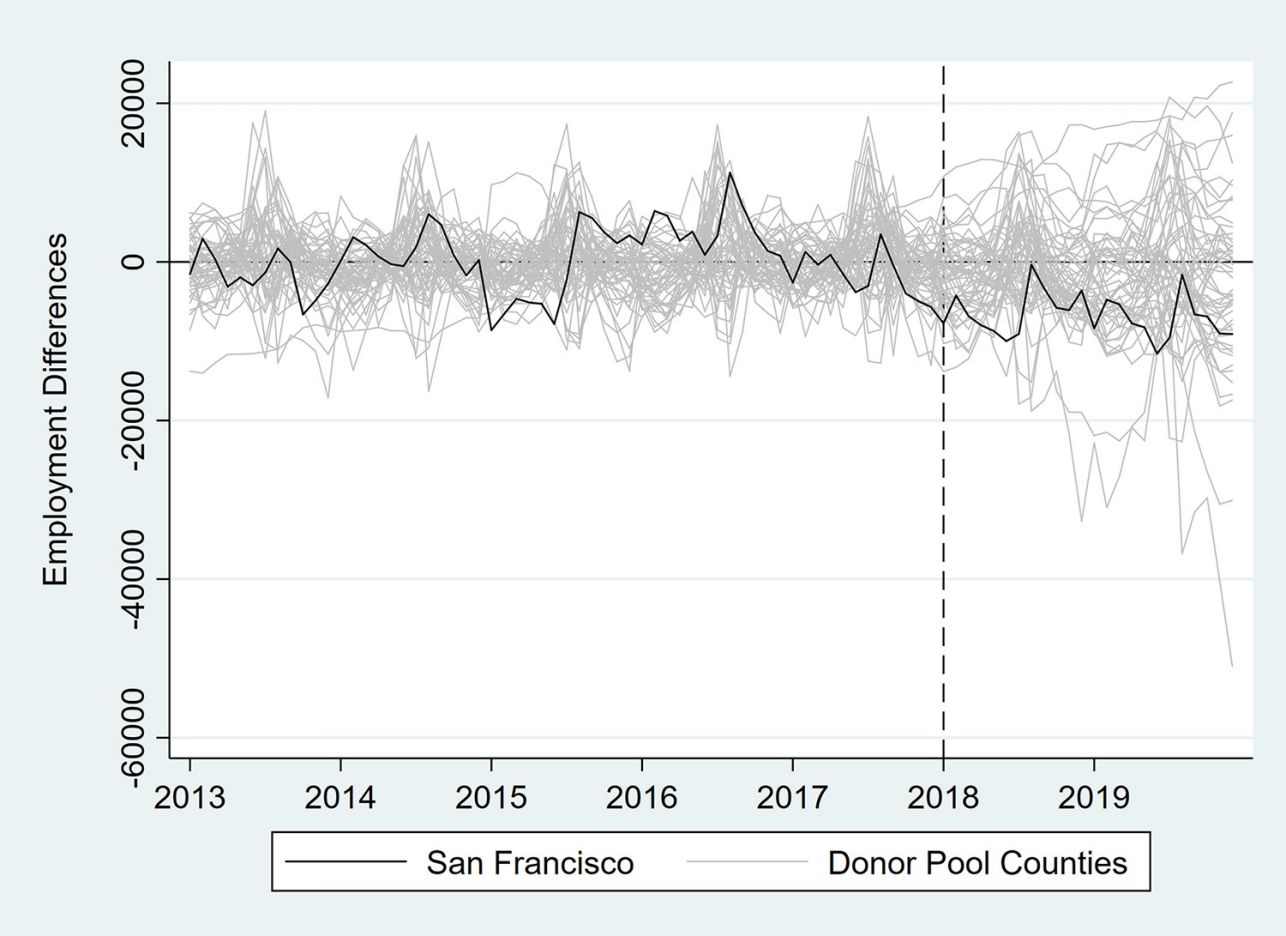
Differences in employment outcomes between each county and its synthetic control for total employment.

**Fig 8:**
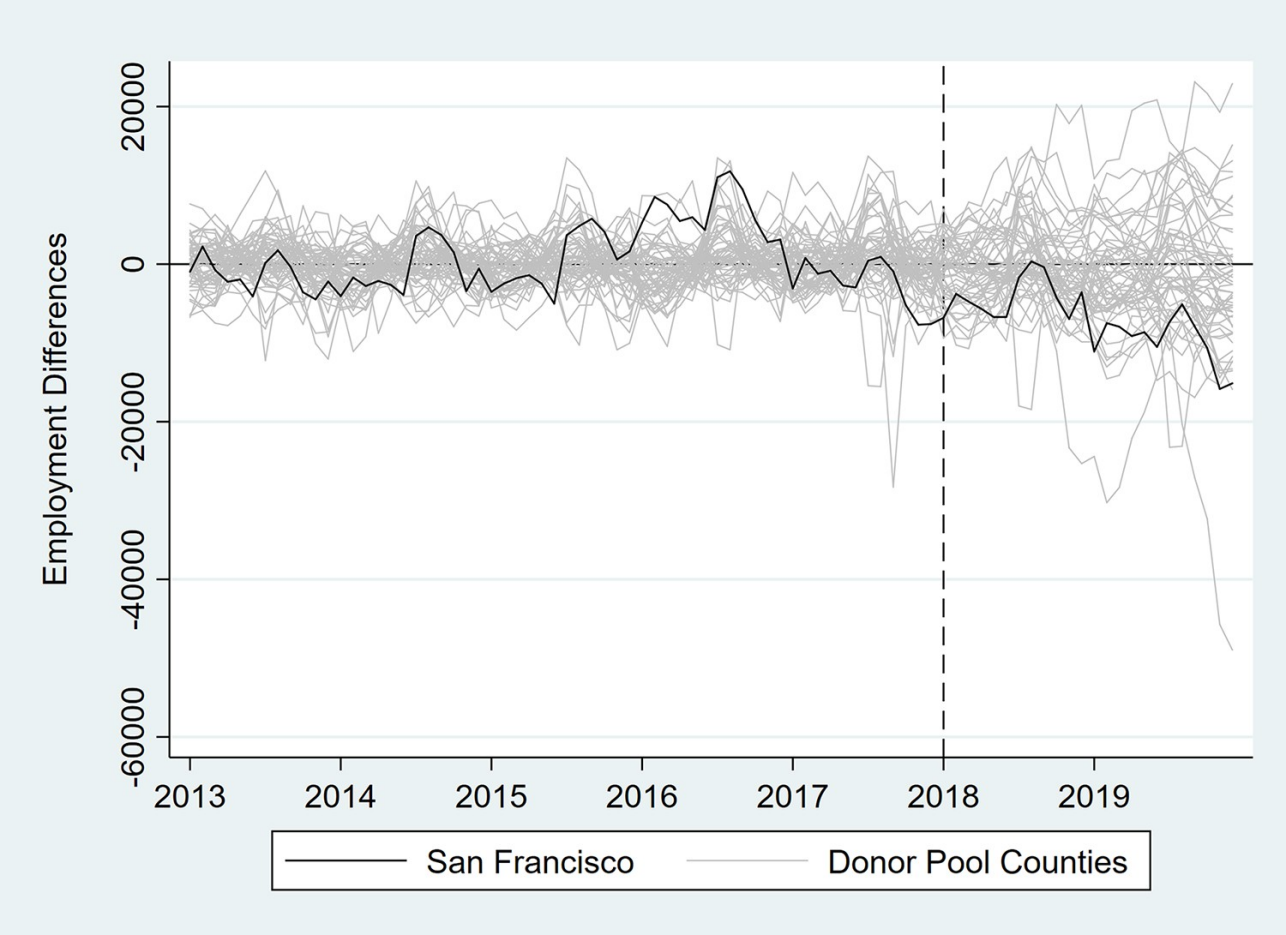
Differences in employment outcomes between each county and its synthetic control for private sector employment.

**Fig 9:**
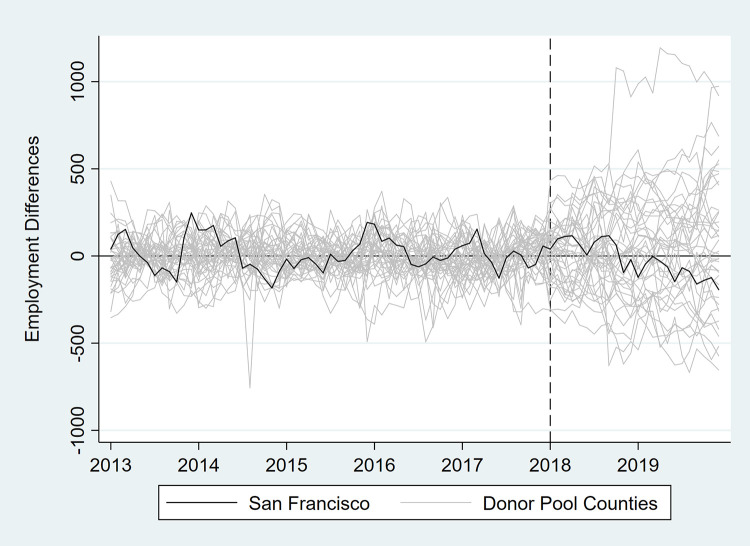
Differences in employment outcomes between each county and its synthetic control for supermarket and other grocery store employment.

**Fig 10:**
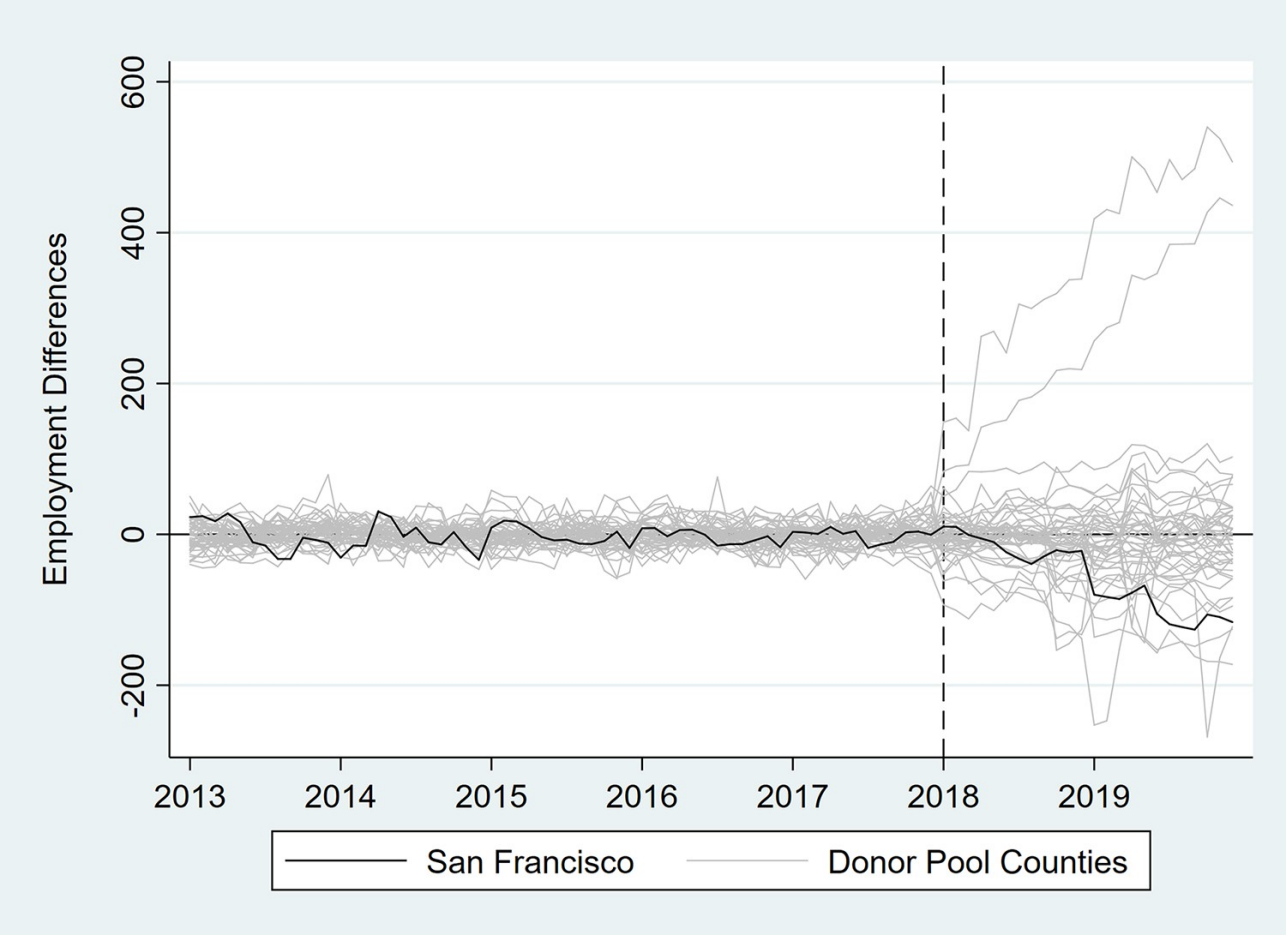
Differences in employment outcomes between each county and its synthetic control for convenience store employment.

**Fig 11:**
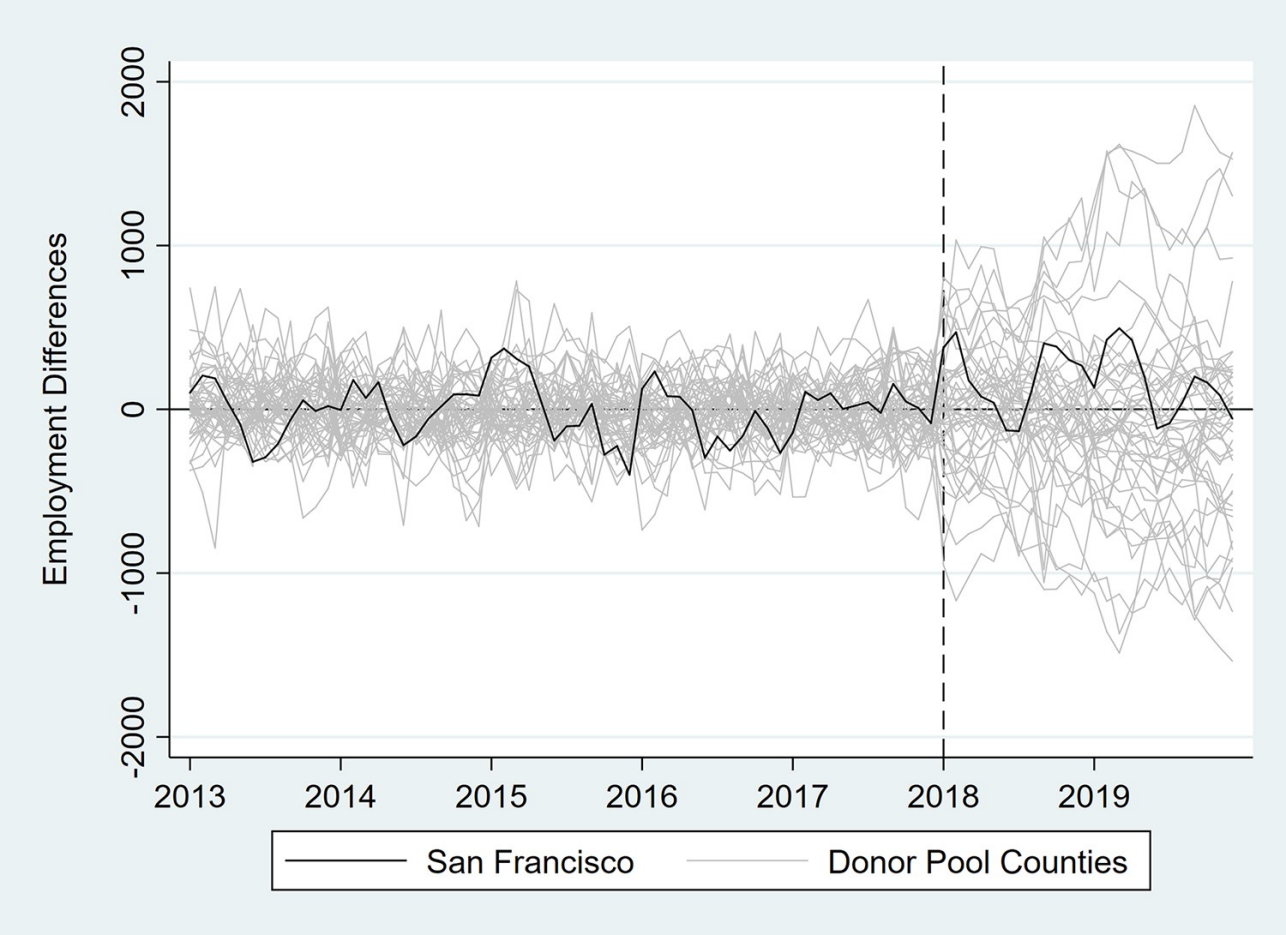
Differences in employment outcomes between each county and its synthetic control for limited-service restaurant employment.

**Fig 12:**
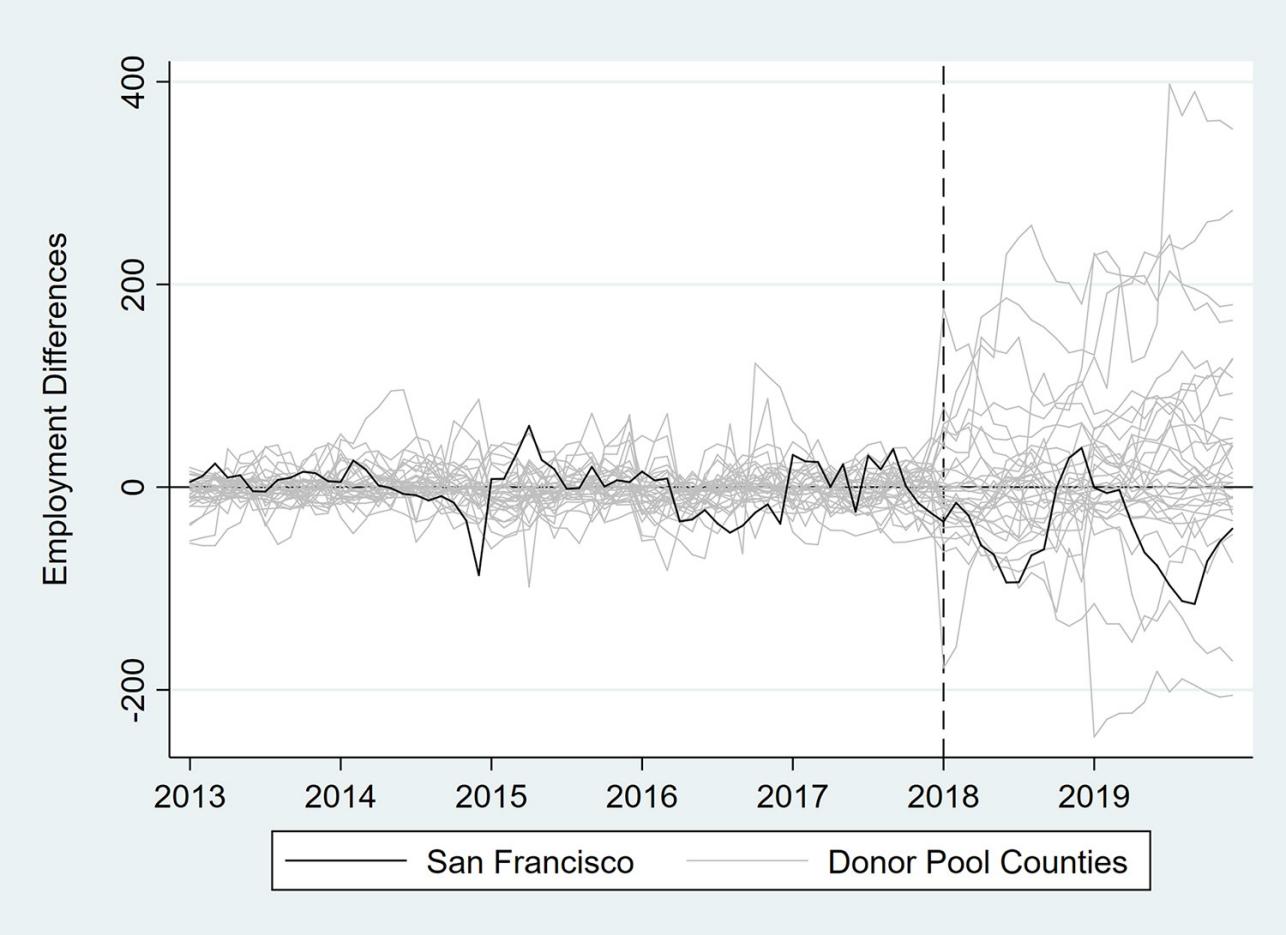
Differences in employment outcomes between each county and its synthetic control for beverage manufacturing employment.

In Table 3 and Figs 7–12, we report on in-space placebo tests that were used to assess the “statistical significance” of the employment impacts of the tax (i.e., post-tax differences in employment between San Francisco and its synthetic control). For each employment outcome, the estimated effect of the SSB tax was compared to placebo effects, which were generated by applying the SCM procedure to the donor pool counties. If the treatment effect was large relative to the placebo effects, then there is more confidence that the effect was not the product of random variation. After the placebo effects were estimated, pseudo “p-values” were calculated as the percentage of placebo effects with post-tax RMSPE (i.e., “average” difference in employment after the tax went into effect) at least as large as that for San Francisco. Donor pool counties with poor pre-tax fit, defined as pre-tax RMSPE more than twice as large as San Francisco’s, were removed from the placebo analysis. Inclusion of these counties would result in pseudo “p-values” that are too conservative because placebo counties with poor pre-treatment fit are also likely to have poor post-treatment fit [17]. None of the estimated treatment effects were “statistically significant” at the 5% level.

**Table 3:** Pseudo p-values and number of counties used in the placebo test for each employment outcome.

Employment outcome	Pseudo p-value	Number of counties
Total	0.26	54
Private sector	0.16	56
Supermarkets and other grocery stores	0.95	40
Convenience stores	0.15	46
Limited-service restaurants	0.57	47
Beverage manufacturing	0.34	32

Finally, we report on the findings from two sensitivity analyses that were conducted to test the robustness of the results to our set of predictors and the pre-tax RMSPE cut-off value required for donor pool counties to be included in the placebo tests. In the first sensitivity analysis, only average employment in 2013, 2015, and 2017 (as opposed to all pre-tax years) were used as lagged outcome variables. Modifying the lagged outcome specification is recommended because these variables have the most influence over donor pool weights. For the second sensitivity, the placebo tests were conducted using only donor pool counties with pre-tax fit (i.e., RMSPE) that was as good as San Francisco’s pre-tax fit or better. In every case, the treatment effects remained “statistically insignificant” at the 5% level. The results of the sensitivity analyses are not shown but are available upon request.

## Discussion

A robust body of empirical evidence is needed to inform policymakers and stakeholders on the potential impacts of SSB taxes on labor market outcomes. To our knowledge, previous empirical tax evaluations have been limited to two jurisdictions—Mexico and Philadelphia, PA [11–13]. While these studies found no negative labor market impacts, their results may not be generalizable to other jurisdictions. This paper provides additional evidence that, in practice, SSB taxes may not result in net job losses or job loss in industries that manufacture and sell beverages. Two years after the San Francisco SSB tax was implemented in January 2018, we found no impacts of the tax on employment in the overall economy, private sector, beverage manufacturing, supermarkets and other grocery stores, convenience stores, or limited-service restaurants. Employment outcomes in San Francisco and the synthetic controls were not found to differ in the post-tax period and these findings were robust to sensitivity analyses.

Findings from this study and other peer-reviewed evaluations differ from predictions made by industry-funded and non-peer-reviewed modeling studies that have been used to argue against SSB taxes [9]. The discrepancy may be explained by the fact that these modeling studies fail to fully account for net impacts of SSB taxes on employment including substitution effects and increased government expenditure from additional tax revenue [9]. The results of the current study are consistent with other peer-reviewed non-industry-funded studies of SSB taxes as well as other policies that aim to reduce consumption of unhealthy foods and beverages. For example, a study of a Chilean food policy package implemented in 2016, which included mandatory front-of-package (FOP) warning labels and restrictions on child marketing and school sales for unhealthy foods and beverages, found no net negative impact on employment [18]. Additionally, our results are consistent with peer-reviewed non-industry-funded research on similar “health taxes” on tobacco and alcohol, which also found no net job losses related to the taxes [7]. Regardless of the specific unhealthy product taxed, the effects of substitution to other goods and services and government spending of tax revenue counteract any negative impacts on employment from reduced purchases of taxed products. Further, because of over-consumption of such products in the absence of a tax, such taxes can reduce consumption-related negative externalities such as medical costs and, additionally, may contribute to improved worker productivity [7].

This study is subject to several limitations. First, only aggregate industry-level data were available, which prevented us from examining differential impacts of the tax within a given industry and by worker characteristics. Second, our analysis does not consider the potential effect of the tax on workers’ hours or wages. Number of hours worked is not included in the QCEW and monthly wage data were not available. However, it is noteworthy that the labor market study of the 2016 Chilean FOP warning labels and child marketing restrictions found no impact of these food-related policies on wages [18]. Third, the study results for San Francisco may not be generalizable to jurisdictions with different distributions of employment across certain industries; for example, in the pre-tax period, the beverage manufacturing industry represented 0.06% of employment in San Francisco compared to 0.15% for the entire U.S. during the same period [19]. Strengths include the use of the SCM, which generated synthetic controls with employment outcomes and labor market-related characteristics similar to those in San Francisco before the tax was implemented, and administrative data that cover over 95% of U.S. jobs. Additionally, the 2-year post-tax period allowed us to assess longer-term impacts of the tax, which is important as it could take time for the labor market to respond to any changes in consumer and government spending.

## Conclusions

The results from this study contribute to emerging evidence on the labor market impacts of SSB taxes. Up to two years after the San Francisco SSB tax was implemented, we find no evidence of job losses overall or in the private sector, nor do we find job losses in the beverage manufacturing, supermarket and other grocery store, convenience store, or limited-service restaurant industries. These findings are consistent with other peer-reviewed SSB tax evaluations of labor market-related unintended consequences and provide additional evidence that such taxes are not associated with reductions in employment.

## Supporting information

S1 Fig Flow chart showing exclusion criteria for donor pools. (TIFF)

S1 Table List of counties and county-level equivalents for each donor pool. (DOCX)

S2 Table Description of synthetic control predictors. (DOCX)

S3 Table Analytical dataset description. (DOCX)

S1 File Analytical dataset. (CSV)
