# Supplementary material for: Employment impacts of the San Francisco sugar-sweetened beverage tax 2 years after implementation
Source: PLoS One. 2021 Jun 2;16(6):e0252094. doi: 10.1371/journal.pone.0252094 (PMC8171954; doi:10.1371/journal.pone.0252094)
Supplement: S1 Table — (DOCX) [file pone.0252094.s002.docx]

**S1 Table. List of counties and county-level equivalents for each donor pool.**

| **County/City, State** | **Total employment** | **Private sector** | **Supermarkets and other grocery stores** | **Convenience stores** | **Limited-service restaurants** | **Beverage manufacturing** |
| --- | --- | --- | --- | --- | --- | --- |
| Alexandria City, VA | X | X | X | X | X |  |
| Allegheny County, PA | X | X | X | X | X |  |
| Arlington County, VA | X | X | X | X | X |  |
| Baltimore City, MD | X | X | X | X | X |  |
| Bexar County, TX | X | X |  |  | X |  |
| Bronx County, NY | X | X | X | X | X | X |
| Charles County, MD |  |  |  |  |  | X |
| Clark County, NV | X | X | X | X | X |  |
| Collin County, TX | X | X | X | X | X | X |
| Comal County, TX |  |  |  |  |  | X |
| Cuyahoga County, OH | X | X | X | X | X | X |
| Dakota County, MN |  |  |  |  |  | X |
| Dallas County, TX | X | X | X | X | X |  |
| Davidson County, TN | X | X | X | X | X |  |
| Dearborn County, IN |  |  |  |  |  | X |
| Denver County, CO | X | X | X | X | X | X |
| Duval County, FL | X | X | X | X | X |  |
| El Dorado County, CA |  |  |  |  |  | X |
| Erie County, NY | X | X | X | X | X | X |
| Essex County, MA |  |  |  |  |  | X |
| Essex County, NJ | X | X | X | X | X |  |
| Fauquier County, VA |  |  |  |  |  | X |
| Franklin County, OH | X | X | X | X | X | X |
| Frederick County, MD |  |  |  |  |  | X |
| Fulton County, GA | X | X | X | X | X |  |
| Hamilton County, OH | X | X | X | X | X | X |
| Harris County, TX | X | X | X | X | X |  |
| Hartford County, CT | X | X | X | X | X |  |
| Hays County, TX |  |  |  |  |  | X |
| Hennepin County, MN | X | X | X | X | X | X |
| Hillsborough County, FL | X | X | X | X | X |  |
| Hudson County, NJ | X | X | X | X | X |  |
| Jackson County, MO | X | X | X | X | X |  |
| Jefferson County, AL | X | X | X | X | X | X |
| Jefferson County, KY | X | X | X | X | X |  |
| Johnson County, KS |  |  |  |  |  | X |
| Kendall County, TX |  |  |  |  |  | X |
| Kent County, MI | X | X | X | X | X |  |
| Kings County, NY | X | X | X | X | X |  |
| Lorain County, OH |  |  |  |  |  | X |
| Los Angeles County, CA | X | X | X | X | X |  |
| Loudoun County, VA |  |  |  |  |  | X |
| Maricopa County, AZ | X | X | X | X | X |  |
| Marion County, IN | X | X | X | X | X | X |
| Mecklenburg County, NC | X | X | X | X | X | X |
| Miami-Dade County, FL | X | X | X | X | X | X |
| Middlesex County, MA |  |  |  |  |  | X |
| Middlesex County, NJ |  |  |  |  |  | X |
| Milwaukee County, WI | X | X | X | X | X |  |
| Monmouth County, NJ |  |  |  |  |  | X |
| Monroe County, NY | X | X | X | X | X |  |
| Multnomah County, OR | X | X | X | X | X |  |
| New York County, NY | X | X | X | X | X |  |
| Norfolk City, VA | X | X | X | X | X |  |
| Norfolk County, MA |  |  |  |  |  | X |
| Oklahoma County, OK | X | X | X | X | X | X |
| Ontario County, NY |  |  |  |  |  | X |
| Orange County, CA | X | X | X | X | X |  |
| Orange County, FL | X | X | X | X | X |  |
| Orange County, NY |  |  |  |  |  | X |
| Orleans Parish, LA | X | X | X | X | X |  |
| Ottawa County, MI |  |  |  |  |  | X |
| Pinellas County, FL | X | X | X | X | X |  |
| Placer County, CA |  |  |  |  |  | X |
| Providence County, RI | X | X | X | X | X |  |
| Queens County, NY | X | X | X | X | X |  |
| Ramsey County, MN | X | X | X | X | X | X |
| Richmond City, VA | X | X | X | X | X |  |
| Richmond County, NY | X | X | X | X | X |  |
| Riverside County, CA | X | X | X | X | X | X |
| Rockingham County, NH |  |  |  |  |  | X |
| Sacramento County, CA | X | X | X | X | X | X |
| Salt Lake County, UT | X | X | X | X | X |  |
| San Benito County, CA |  |  |  |  |  | X |
| San Diego County, CA | X | X | X | X | X |  |
| Shelby County, AL |  |  |  |  |  | X |
| Shelby County, TN | X | X | X | X | X |  |
| St. Louis City, MO | X | X | X | X | X |  |
| St. Tammany Parish, LA |  |  |  |  |  | X |
| Suffolk County, MA | X | X | X | X | X |  |
| Tarrant County, TX | X | X | X | X | X |  |
| Travis County, TX | X | X | X | X | X |  |
| Union County, NJ | X | X | X | X | X |  |
| Virginia Beach City, VA | X | X | X | X | X |  |
| Wake County, NC | X | X | X | X | X |  |
| Washington, D.C. | X | X | X | X | X |  |
| Waukesha County, WI |  |  |  |  |  | X |
| Westchester County, NY |  |  |  |  |  | X |
| Westmoreland County, PA |  |  |  |  |  | X |
| Wayne County, MI | X | X | X | X | X |  |
| Yamhill County, OR |  |  |  |  |  | X |
| Yates County, NY |  |  |  |  |  | X |
| Yolo County, CA |  |  |  |  |  | X |
