# Supplementary material for: Employment impacts of the San Francisco sugar-sweetened beverage tax 2 years after implementation
Source: PLoS One. 2021 Jun 2;16(6):e0252094. doi: 10.1371/journal.pone.0252094 (PMC8171954; doi:10.1371/journal.pone.0252094)
Supplement: S2 Table — (DOCX) [file pone.0252094.s003.docx]

**S2 Table. Description of synthetic control predictors.**

| **Variable** | **Data source(s)** | **Computation** |
| --- | --- | --- |
| County-level |  |  |
| Unemployment rate | Bureau of Labor Statistics (BLS): Local Area Unemployment Survey | Averaged over 2013 to 2017 based on annual data |
| Gross domestic product ($ 000s) | Bureau of Economic Analysis (BEA): Local Area Gross Domestic Product | Averaged over 2013 to 2017 based on annual data |
| Adjusted personal income per capita | BEA: Local Area Personal Income  Economic Policy Institute’s Family Budget Calculator: Annual Total Income needed for two adults and two children to attain adequate standard of living | Personal income per capital averaged over 2013 to 2017 based on annual data and divided by single estimate of cost of living in 2017 |
| Population density per square mile | Census Bureau: 2010 Decennial Census | Single estimate for 2010 |
| Total population | Census Bureau: 2013-2017 American Community Survey 5-Year Data Profile | Single estimate for 2013 to 2017 |
| Prime-age workers (25-54) (%) | Census Bureau: 2013-2017 American Community Survey 5-Year Data Profile | Single estimate for 2013 to 2017 |
| Industry-specific |  |  |
| Employment, 2013 | BLS: Quarterly Census of Employment and Wages (QCEW) | Averaged over 2013 based on monthly data |
| Employment, 2014 | BLS: QCEW | Averaged over 2014 based on monthly data |
| Employment, 2015 | BLS: QCEW | Averaged over 2015 based on monthly data |
| Employment, 2016 | BLS: QCEW | Averaged over 2016 based on monthly data |
| Employment, 2017 | BLS: QCEW | Averaged over 2017 based on monthly data |
| Industry share (%) | BLS: QCEW | A given industry’s average employment from 2013 to 2017 divided by average total employment from 2013 to 2017 based on monthly data |
| Number of establishments | BLS: QCEW | Averaged over 2013 to 2017 based on quarterly data |
