# Supplementary material for: Employment impacts of the San Francisco sugar-sweetened beverage tax 2 years after implementation
Source: PLoS One. 2021 Jun 2;16(6):e0252094. doi: 10.1371/journal.pone.0252094 (PMC8171954; doi:10.1371/journal.pone.0252094)
Supplement: S3 Table — (DOCX) [file pone.0252094.s004.docx]

**S3 Table. Analytical Dataset Description.**

| **Variable** | **Description** |
| --- | --- |
| area_fips | Area Federal Information Processing Standard code |
| countyname | County or county-level equivalent name |
| stateabr | State abbreviation |
| year | Year |
| month | Month |
| total_emp | Total employment |
| private_emp | Private sector employment |
| bev_emp* | Beverage manufacturing employment |
| super_emp* | Supermarket and other grocery store employment |
| conven_emp* | Convenience store employment |
| ff_emp* | Limited-service restaurant employment |
| total_estab | Total number of establishments |
| private_ estab | Number of private sector establishments |
| bev_ estab | Number of beverage manufacturing establishments |
| super_ estab | Number of supermarket and other grocery store establishments |
| conven_ estab | Number of convenience store establishments |
| ff_ estab | Number of limited-service restaurant establishments |
| gdp_2013 | Gross domestic product ($ 000s), 2013 |
| gdp_2014 | Gross domestic product ($ 000s), 2014 |
| gdp_2015 | Gross domestic product ($ 000s), 2015 |
| gdp_2016 | Gross domestic product ($ 000s), 2016 |
| gdp_2017 | Gross domestic product ($ 000s), 2017 |
| pop_density | Population density per square mile |
| inc_2013 | Personal income per capita, 2013 |
| inc_2014 | Personal income per capita, 2014 |
| inc_2015 | Personal income per capita, 2015 |
| inc_2016 | Personal income per capita, 2016 |
| inc_2017 | Personal income per capita, 2017 |
| total_pop | Total population |
| prime_age | Percentage of prime-age workers (25-54) |
| unemp_rate_2013 | Unemployment rate, 2013 |
| unemp_rate_2014 | Unemployment rate, 2014 |
| unemp_rate_2015 | Unemployment rate, 2015 |
| unemp_rate_2016 | Unemployment rate, 2016 |
| unemp_rate_2017 | Unemployment rate, 2017 |
| nchs_code | National Center for Health Statistics’ 2013 Urban-Rural Classification Scheme code (1: large central metro, 2: large fringe metro) |
| fam_budget | Economic Policy Institute’s Family Budget Calculator annual total income needed for two adults and two children to attain adequate standard of living |

* A code of “.s” indicates the value was suppressed.
